# Identification of seven Zingiberaceous species based on comparative anatomy of microscopic characteristics of seeds

**DOI:** 10.1186/1749-8546-9-10

**Published:** 2014-03-08

**Authors:** Meng-hua Wu, Wei Zhang, Ping Guo, Zhong-zhen Zhao

**Affiliations:** 1 School of Chinese Medicine, Hong Kong Baptist University, Jockey Club SCM Building, 7 Baptist University Road, Kowloon Tong, Hong Kong, China; 2 Guangdong Pharmaceuticals Holdings Co., Ltd., Guangzhou, Guangdong, China; 3 Guangzhou Institute for Drug Control, 23 Xizeng Road, Liwan District, Guangzhou, Guangdong, China

## Abstract

**Background:**

The fruits and seeds of *Alpinia galanga* (L.) Willd., *Alpinia katsumadai* Hayata, *Alpinia zerumbet* (Pers.) Burtt. & Smith, *Amomum kravanh* Pierre ex Gagnep., *Amomum subulatum* Roxb., *Amomum tsao-ko* Crevost et Lemaire, and *Elettaria cardamomum* (L.) Maton from *Alpinia*, *Amomum,* and *Elettaria* genera in the Zingiberaceae family are difficult to distinguish between each other. This study aims to identify the seeds of these seven species from Zingiberaceae family based on comparative anatomy of microscopic characteristics.

**Methods:**

We compared the morphological structures of seed coats by observing the microscopic characteristics of seeds in transverse sections. We described the macroscopic characteristics of seeds in detail.

**Results:**

The seeds of these three genera could not be identified to the species level based on their macroscopic features. However, based on the anatomical features of the seed coat observed in transverse sections, a dichotomous key for these seven species was feasible.

**Conclusion:**

Seven species in the Zingiberaceae family could be identified based on comparative anatomy of microscopic characteristics of transverse section of seed.

## Background

Most fruits of *Alpinia*, *Amomum*, and *Elettaria* in Zingiberaceae family are used in cooking and medicine in China [1]. In China, 51 species belong to the *Alpinia* genus, 39 species to the *Amomum* genus [2], and 1 species to the *Elettaria* genus [3]. The fruits of approximately 20 species in the Zingiberaceae were documented with text and drawing in detail in literature [3]. The species of Zingiberaceae was usually identified by the seed and fruit characteristics.

*Alpinia galanga* (L.) Willd., *Alpinia katsumadai* Hayata, *Alpinia zerumbet* (Pers.) Burtt. & Smith, *Amomum kravanh* Pierre ex Gagnep., *Amomum subulatum* Roxb., *Amomum tsao-ko* Crevost et Lemaire and *Elettaria cardamomum* (L.) Maton are three different genera in the Zingiberaceae family. For all of these species, a fruit is a capsule with three locules that contain numerous seeds [4]. Their Chinese common names always include “*doukou*” as a suffix and their English common names sometimes include “cardamom” [1]. Because of their similar appearance and similar common names, these fruits have been confused with each other since ancient times [5].

Several studies focused on the anatomic structures and systematic significance of roots and leaves of plants from the Zingiberaceae. The morphological characteristics of roots and leaves were studied for the theoretical basis for determining the phylogenetic and evolutionary relationships among species and genera [6-8]. As seeds play a major role in providing continuity, as well as the potential for genetic variation, between successive generations of seed plants [9], the morphological structures of seeds, especially the seed coat, also have unique and relatively stable characteristics [10,11]. This study aims to identify these seven species of Zingiberaceae family based on comparative anatomy of microscopic characteristics of seeds in transverse section.

## Methods

Three batches of fruits and seeds of *A. galanga, A. katsumadai*, *A. zerumbet*, *A. kravanh, A. subulatum*, *A. tsao-ko*, and *E. cardamomum* were collected from Chinese herbal markets and spice retailers at Hehuachi in Chengdu, Shapingba in Chongqing, Qingping in Guangzhou, Kowloon City Market and Ko Shing Street in Hong Kong, and from an Indian spice market in Old Delhi. All samples were identified to the species level by Prof. Zhongzhen Zhao of Hong Kong Baptist University and Prof. Delin Wu of South China Botanical Garden based on traditional macroscopic identification which must rely on enough experience [3]. Samples and voucher specimens are deposited at the Bank of China (Hong Kong) Chinese Medicines Centre at Hong Kong Baptist University.

Macroscopic identification was conducted as described elsewhere [12]. We noted the appearance, color, odor, and taste of the samples, and took color digital photographs.

Seeds were fixed in FAA 70 for a minimum of 24 h. After fixing, the seeds were passed through graded solutions of ethanol (50%, 70%, 80%, 90%; absolute grade) (Sasma, The Netherlands) and xylene (50%; absolute grade) (Lab-Scan, Bangkok, Thailand), embedded in paraffin wax (Unichem Ltd. Chessington, UK) using the technique described by Ruzin [13], and finally cut into 15-μm-thick sections using a rotary microtome (Thermo Shandon, Cheshire, UK). Sections were stained with Safranin-T (Fluka, Brazil) and fast green FCF solution (Sigma-Aldrich, St Louis, MO, USA). Finally, they were sealed with Canada balsam (Sigma-Aldrich). At least ten different transverse sections from each sample were prepared and observed under Axioplan 2 and Axiophot 2 universal microscopes equipped with a reflector Axiophot photo module (Zeiss Group, Jena, Germany) with a Leica direct current (DC) camera.

Essential oils were isolated from seed and fruit samples by hydrodistillation to study the relationship between essential oil yield and the size of the oil cell layer for each species [4]. The collected plant materials were macerated with a copper mortar and pestle. The broken samples were subjected to 3 hours of hydrodistillation by a Clevenger-type apparatus. The volume of essential oils obtained was read directly from the apparatus.

## Results

### Macroscopic features of fruits and seeds

**Figure 1:**
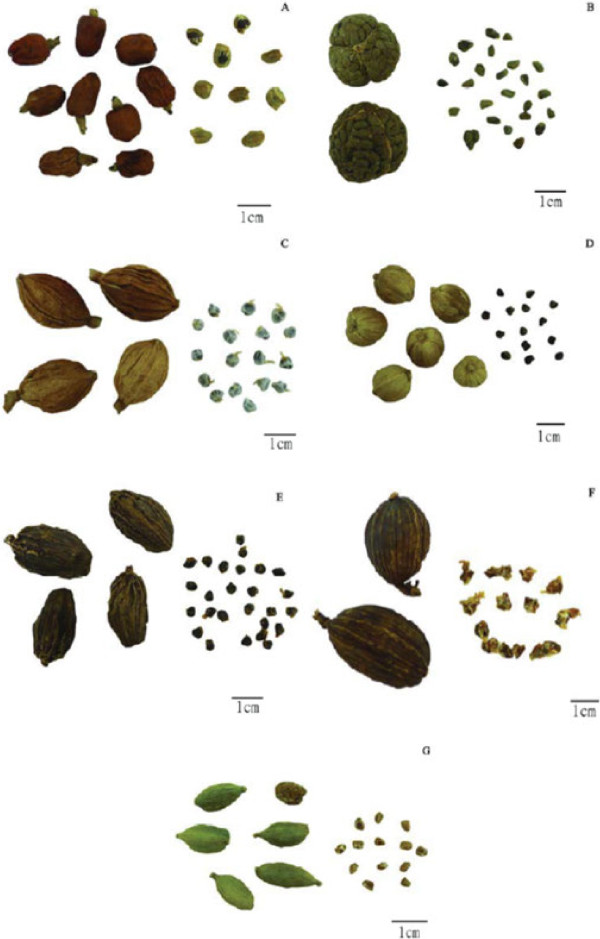
**Photographs of fruits and seeds of seven Zingiberaceous species. A**. *Alpinia galanga*; **B**. *Alpinia katsumadai*; **C**. *Alpinia zerumbet*; **D**. *Amomum kravanh*; **E**. *Amomum subulatum*; **F**. *Amomum tsao-ko*; **G**. *Elettaria cardamomum.*

*A. galanga*: fruit a capsule, oblong, slightly narrow in the middle, with three locules, two seeds in each locule; pericarp dark red or brown, shrunken, or smooth sometimes, apex with yellowish-white tubular persistent calyx. Seeds oblate outside, blackish-brown, covered with yellowish-white membranous aril (Figure 1-A). Odor: aromatic; taste: pungent.

*A. katsumadai*: seed masses spheroidal, three locules divided evenly by septa, numerous seeds agglutinated closely in each locule. Seeds ovoid-polyhedral, covered with grayish-brown membranous aril, raphe occurring as a longitudinal furrow (Figure 1-B). Odor: aromatic; taste: pungent and slightly bitter.

*A. zerumbet*: fruit a capsule, spindle-shaped, with three locules, more than ten seeds in each locule, arranged loosely; pericarp yellowish-white to yellowish-brown, with longitudinal winged ribs, apex with tubular persistent calyx. Seeds polyhedral, covered with white membranous aril (Figure 1-C). Odor: aromatic; taste: slightly pungent.

*A. kravanh*: fruit a capsule, subspherical, with three locules, seven to ten seeds in each locule, pericarp yellowish-white to yellowish-brown, with three relatively deep longitudinal furrows, apex possessing a prominent stylopodium. Seeds polyhedral, dark brown, with remains of aril (Figure 1-D). Odor: aromatic; taste: pungent and cool, slightly camphor-like.

*A. subulatum*: fruit a capsule, long ellipsoid, slightly curved, flat on one side, with three locules, approximately 20 seeds in each locule; pericarp grayish-brown to brown, with longitudinally winged ribs near the calyx apex, apex with long tubular persistent calyx. Seeds ovoid-polyhedral, dark brown, slightly adhesive, with little remains of aril (Figure 1-E). Odor: aromatic, smoky-like; taste: slightly pungent.

*A. tsao-ko*: fruit a capsule, long ellipsoid, three-obtuse-ridged, with three locules, approximately 20 seeds in each locule; pericarp grayish-brown to brown, with longitudinal furrows and ribs, and a fruit stalk at the base. Seeds conicalpolyhedral, reddish-brown, covered with grayish-white membranous aril, slightly shrunken (Figure 1-F). Odor: aromatic; taste: pungent and slightly bitter.

*E. cardamomum*: fruit a capsule, long ellipsoid, three-obtuse-ridged, with three locules, two to seven seeds in each locule; pericarp green to yellowish-green, with longitudinal furrows and ribs arranged densely, slightly dehiscent at the base. Seeds oblong-ovoid, pale orange, covered with colorless membranous aril (Figure 1-G). Odor: strong aromatic; taste: pungent and slightly bitter.

### Microscopic features of seed transverse sections

**Figure 2:**
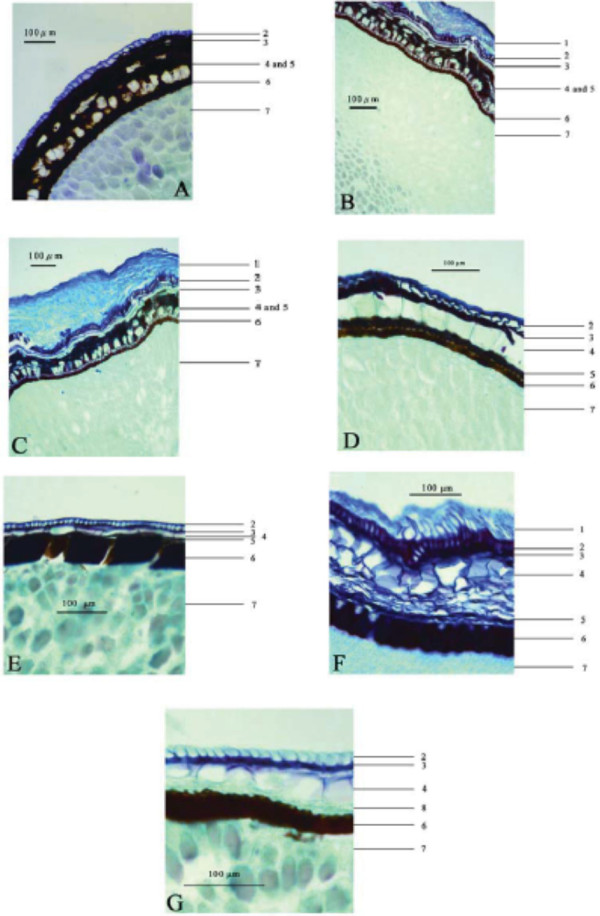
**Micrographs showing transverse sections of seed coats of seven Zingiberaceous species. A**. *Alpinia galanga*; **B**. *Alpinia katsumadai*; **C**. *Alpinia zerumbet*; **D**. *Amomum kravanh*; **E**. *Amomum subulatum*; **F**. *Amomum tsao-ko*; **G**. *Elettaria cardamomum.* 1. Aril cells; 2. Epidermal cells of testa; 3. Hypodermal cells of testa; 4. Oil cells; 5. Pigment layer; 6. Palisade sclerenchymatous cells of endotesta; 7. Perisperm cells; 8. Obliterated cells.

*A. galanga*: Epidermis of testa consisting of one layer of cells, longitudinally elongated, wall relatively thickened. Hypodermis consisting of one layer of cells, tangentially elongated, with brown-red pigment. Pigment layer consisting of several layers of brown cells, scattered with one to three layers of subrounded oil cells. Endotesta consisting of one layer of palisade sclerenchymatous cells, brown-red, with heavily thickened inner and lateral walls, lumina small, about one-fifth to one-fourth the thickness of sclerenchymatous cells (Figure 2-A).

*A. katsumadai*: Aril consisting of seven to ten layers of cells, tangentially elongated, with slightly thickened walls. Epidermis of testa consisting of one layer of cells, longitudinally elongated, wall relatively thickened. Hypodermis consisting of two to four layers of cells, tangentially elongated. Pigment layer consisting of several layers of brown cells, scattered with one to three layers of subrounded oil cells. Endotesta consisting of one layer of palisade sclerenchymatous cells, brown-red, with heavily thickened inner and lateral walls, lumina about half the thickness of sclerenchymatous cells (Figure 2-B).

*A. zerumbet*: Aril consisting of four to seven layers of cells, rounded or longitudinally elongated, with slightly thickened walls. Epidermis of testa consisting of one layer of cells, longitudinally elongated, wall relatively thickened. Hypodermis consisting of three to four layers of cells, tangentially elongated. Pigment layer consisting of several layers of brown cells, scattered with one to two layers of subrounded oil cells. Endotesta consisting of one layer of palisade sclerenchymatous cells, brown-red, with heavily thickened inner and lateral walls, lumina about half the thickness of sclerenchymatous cells (Figure 2-C).

*A. kravanh*: Epidermis of testa consisting of one layer of cells, longitudinally elongated, wall relatively thickened, especially the outside. Hypodermis consisting of one layer of cells, with brown-red pigment. Oil cells one layered, large, subsquare. Pigment layer consisting of several layers of brown cells. Endotesta consisting of one layer of palisade sclerenchymatous cells, brown-red, with heavily thickened inner and lateral walls, lumina small, about one-fifth to one-fourth the thickness of sclerenchymatous cells (Figure 2-D).

*A. subulatum*: Epidermis of testa consisting of one layer of cells, subrounded, wall relatively thickened, especially the outside. Hypodermis consisting of one layer of cells, with brown-red pigment. Oil cells one layered, tangentially elongated. Pigment layer consisting of several layers of brown cells. Endotesta consisting of one layer of palisade sclerenchymatous cells, brown-red, with heavily thickened inner and lateral walls, lumina very small, about one-twentieth to one-tenth the thickness of sclerenchymatous cells, border indistinct (Figure 2-E).

*A. tsao-ko*: Aril consisting of four to seven layers of cells, longitudinally elongated, with slightly thickened walls. Epidermis of testa consisting of one layer of cells, longitudinally elongated, wall relatively thickened. Hypodermis consisting of one layer of cells, tangentially elongated, with brown-red pigment. Oil cells one layered, subsquare or rectangular. Pigment layer consisting of several layers of brown cells, shrunken. Endotesta consisting of one layer of palisade sclerenchymatous cells, brown-red, with heavily thickened inner and lateral walls, lumina about one-fourth to one-third the thickness of sclerenchymatous cells (Figure 2-F).

*E. cardamomum*: Epidermis of testa consisting of one layer of cells, longitudinally elongated. Hypodermis consisting of one layer of cells, with brown-red pigment, tangentially elongated. Oil cells one layered, large, subsquare. Obliterated cells single-layered, with no pigment. Endotesta consisting of one layer of palisade sclerenchymatous cells, brown-red, with heavily thickened inner and lateral walls, lumina about one-eighth to one-tenth the thickness of sclerenchymatous cells (Figure 2-G).

### Yield of essential oils

The average yield of the essential oil of *A. galanga* fruits, *A. katsumadai* seeds, *A. zerumbet* fruits, *A. kravanh* fruits, *A. subulatum* fruits, *A. tsao-ko* fruits, and *E. cardamomum* fruits was 0.2 mL, 1.0 mL, 1.0 mL, 5.0 mL, 1.8 mL, 1.3 mL, and 7.0 mL per 100 g of sample, respectively (*n* = 3). The seeds of three species from the *Alpinia* genus contained no more than 1% (vol/weight) essential oil, while those of three species from the *Amomum* genus and one species from *Elettaria* genus yielded more than 1% (vol/weight) essential oil.

## Discussion

The macroscopic features of Zingiberaceous fruits from species in the *Alpinia*, *Amomum*, and *Elettaria* genera can be summarized as follows: fruit a capsule, axile placentation, seeds with arils and numerous seeds attached to the central axis of an ovary with three locules. There is no distinct differentiation among these three genera based on the macroscopic features of the fruits and seeds.

Our results showed that the microscopic features of the seed coat, as observed in transverse sections, can be used to distinguish these three genera (Table 1). The hypodermis of the testa of seeds from species of the *Alpinia* genus has one or more layers of cells without pigment, while the hypodermis of testa of seeds from the *Amomum* and *Elettaria* genera has only one layer of cells with pigment. The distribution of oil cell layers and pigment layers differ among the three genera. Several layers of pigmented cells scattered with one to three layers of oil cells are features of seeds in the *Alpinia* genus. The yield of essential oil from these seeds was not more than 1% (vol/weight). Seeds in the *Amomum* genus have one layer of relatively large oil cells with several layers of pigmented cells at their inner sides. The typical essential oil yield from these seeds was more than 1% (vol/weight). Seeds in the *Elettaria* genus have one layer of large oil cells with one layer of obliterated cells at the inner side. These seeds had a significantly greater yield of essential oil, up to 7% (vol/weight). The volume of essential oil yielded from seeds will be useful for rapid genus identification. An anatomic key is listed in Table 2.

**Table 1:** Microscopic characteristics of seed coats of seeds of seven Zingiberaceous species as observed in transverse sections

**Species**	**Epidermis of testa (layer)**	**Hypodermis of testa (layer)**	**Pigmentation of hypodermis of testa**	**Oil cell layers**	**Pigment layers**	**Obliterated cells layer**	**Ratio of thickness of lumina to endotesta palisade sclerenchymatous cells**
*Alpinia galanga*	1	1	No	1–3 layers, scattered in pigment layer	Several layers of brown cells	None	1/5–1/4
*Alpinia katsumadai*	1	2–4	No	1–3 layers, scattered in pigment layer	Several layers of brown cells	None	Approx. 1/2
*Alpinia zerumbet*	1	3–4	No	1–2 layers, scattered in pigment layer	Several layers of brown cells	None	Approx. 1/2
*Amomum kravanh*	1	1	Yes	1	Several layers, cell wall not distinct	None	1/5–1/4
*Amomum subulatum*	1	1	Yes	1	Several layers, cell wall not distinct	None	1/20–1/10
*Amomum tsao-ko*	1	1	Yes	1	Several layers, cell wall distinct and shrunken	None	1/4–1/3
*Elettaria cardamomum*	1	1	Yes	1	None	1 layer	1/8–1/10

**Table 2:** **Dichotomous key to seven species in** ***Alpinia*,** ***Amomum,*** **and** ***Elettaria*** **genera based on anatomical characteristics of seeds**

1. Fruit a capsule, axile placentation, seeds with arils and numerous seeds attached to the central axis of an ovary with three locules	
2a. Hypodermis of testa comprising one layer of cells with pigment	
3a. Inner side of oil cell layer with several layers of pigmented cells	
4a. Pigmented cell wall not distinct	
5a. Ratio of thickness of lumina to endotesta palisade sclerenchymatous	
cells 1:5–1:4	1. *Amomum kravanh*
5b. Ratio of thickness of lumina to endotesta palisade sclerenchymatous	
cells 1:10–1:20	2. *Amomum subulatum*
4b. Pigmented cell wall distinct and shrunken	3. *Amomum tsao-ko*
3b. Inner side of oil cell layer with one layer of obliterated cells	4. *Elettaria cardamomum*
2b. Hypodermis of testa comprising one or more layers of cells without pigment, several layers of pigment cells scattered with oil cells	
6a. Ratio of thickness of lumina to endotesta palisade sclerenchymatous	
cells approx. 1:2	
7a. Oil cells in 1–3 layers	5. *Alpinia katsumadai*
7b. Oil cells in 1–2 layers	6. *Alpinia zerumbet*
6b. Ratio of thickness of lumina to endotesta palisade sclerenchymatous	
cells 1:5–1:4	7. *Alpinia galanga*

The position of inflorescences and the shape of bracteoles are the main characteristics distinguishing *Alpinia*, *Amomum*, and *Elettaria* genera. The microscopic features of seed coats, as observed in transverse sections, and the yield of essential oils, provide a taxonomic basis for distinguishing these species. Even though the seeds have similar morphological structures, it appears that different characteristics of each layer of the testa can distinguish each species among these three genera. The microscopic identification of seed coat characteristics in transverse sections is simple and reliable; and could be used to identify culinary spices and herbal medicines.

## Conclusion

Seven species in the Zingiberaceae family could be identified based on comparative anatomy of microscopic characteristic of seeds in transverse section.

## Abbreviations

CMM: Chinese materia medica.

## Competing interests

The authors declare that they have no competing interests.

## Authors’ contributions

MHW designed the study. MHW and WZ performed data analysis and collected herbal materials. MHW wrote the manuscript. PG and ZZZ monitored the conduct of the study and revised the manuscript. All authors have read and approved the final manuscript.
